# A Decade of Follow-Up to Assess the Risk of Recurrence and Surgery after a First Episode of Uncomplicated Left-Sided Diverticulitis

**DOI:** 10.3390/jcm13195854

**Published:** 2024-09-30

**Authors:** Dario Carletta, Sotirios Georgios Popeskou, Francesco Mongelli, Nicole Murgante, Matteo Di Giuseppe, Francesco Proietti, Martin Hübner, Dimitrios Christoforidis

**Affiliations:** 1 Dipartimento di Chirurgia Viscerale, Ospedale Regionale di Lugano, Via Tesserete 46, 6900 Lugano, Switzerland; sotiriosgeorgios.popeskou@eoc.ch (S.G.P.); dimitrios.christoforidis@eoc.ch (D.C.); 2 Dipartimento di Chirurgia Viscerale, Ospedale Regionale di Bellinzona e Valli, Via Ospedale 12, 6500 Bellinzona, Switzerland; 3 Dipartimento di Chirurgia Viscerale, Ospedale Regionale di Locarno, Via dell’Ospedale 1, 6600 Locarno, Switzerland; 4 Service de Chirurgie Viscérale, Centre Hospitalier Universitaire Vaudois, Rue du Bugnon 46, 1005 Lausanne, Switzerland

**Keywords:** diverticulitis, colorectal surgery, sigmoidectomy, surgery, laparoscopy

## Abstract

**Background and aims**: Acute uncomplicated diverticulitis (UD) of the left colon is common and mostly benign. Due to controversy over the definition of UD and the lack of adequate follow-up in most studies, good quality data to predict long-term outcomes after a first episode of UD are missing. The aim of this study was to assess the long-term risk for adverse outcomes after a first episode of UD. **Methods**: All consecutive patients with a CT-scan-documented first episode of acute UD (staged “uncomplicated” according to ESCP guidelines and/or modified Hinchey stages 0-1a, and/or CDD 1-2a) between January 2010 and June 2013 were included in the study. CT scans and clinical records were retrospectively reviewed. The primary endpoint was overall recurrence; the secondary endpoint was surgery for diverticular disease. **Results**: One hundred and five patients were included in the study with a median follow-up of 116.4 (4.9–154.7) months. Of these, 51 (48.5%) patients had a recurrence, 11 (10.4%) had 4 or more episodes. Twenty-one (20%) patients underwent sigmoidectomy, all in an elective setting, mostly due to multiple recurrent episodes. Male gender was the only independent risk factor for surgery (OR (95%CI): 0.301 (0.109–0.834), *p* = 0.021). Classification systems did not predict recurrence, but stage CDD 1a was protective for surgery (OR (95%CI): 0.201 (0.042–0.957), *p* = 0.044). **Conclusions**: After a decade of follow-up, almost half the patients experienced at least one recurrent episode after UD, higher than previously thought. None of those patients required emergency surgery, but one in five patients, mostly men, underwent elective sigmoidectomy for multiple recurrent episodes.

## 1. Introduction

The definition of uncomplicated diverticulitis (UD) is variable and prone to interpretation despite the fact that this clinical entity accounts for approximately 85% of acute cases [1,2]. Acute diverticulitis is generally considered complicated in the presence of bowel perforation with free peritoneal air and liquid, peritonitis, or abscesses, usually requiring surgical or radiological intervention. UD differs among classifications [3,4,5,6,7,8]. Although the typical UD consists of pericolic inflammation and mild colonic wall thickening, patients with small paracolic collections or the presence of a small quantity of air bubbles are also typically managed conservatively, usually with antibiotics in everyday practice with success rates close to 100%. Yet, depending on the classification system used, they could be interpreted as uncomplicated or complicated. A pragmatic classification should help the clinician choose the appropriate management strategy for the acute episode with or without antibiotics, as an in- or outpatient, and possibly predict the disease’s long-term course.

According to the current literature, after a first uncomplicated episode treated conservatively, approximately 30% will remain asymptomatic [9], 10–25% [9,10] will have recurrent episodes and up to 20–35% [9] will continue to have some form of chronic abdominal pain. In about 4–10% [11], the pain may be due to smoldering diverticulitis. A study with a 10-year follow up found recurrence after a first episode to be 22%, and 55% after a second one [12].

The aim of this study was to investigate characteristics that could better differentiate this heterogeneous population of patients with “UD”, that could predict adverse outcomes and more specifically, long term recurrence after a sufficiently long follow-up.

## 2. Materials and Methods

### 2.1. Study Design and Patient Selection

All authors had access to the study data and reviewed and approved the final manuscript. The ethics committee approved our study (BASEC ID: 2020-02386). A retrospective analysis of all patients diagnosed with a first episode of left-sided diverticulitis documented by a CT scan at the Lugano Regional Hospital in Switzerland spanning from 1 January 2010 to 30 June 2013 was performed. Only patients diagnosed with a CT scan and no other diagnostic methods were included and only those who had no prior anamnesis or documentation for a previous diverticulitis episode. All CT-scan reports that contained the keyword “diverticulitis” were included in the study. The CT scan images were then double-checked, by four board certified radiologists to confirm the diagnosis and in order to increase accuracy; CT scans were classified according to three different classifications: The ESCP, the modified Hinchey and the CDD.

We included all patients with a first episode of left-sided diverticulitis, staged ESCP “uncomplicated”, mHinchey stages 0-1a, and CDD 1-2a. We included CDD stage 2a patients (micro-abscess < 1 cm) despite being considered “complicated”, given their conservative management and potential different evolution concerning the risk of recurrence and/or surgery.

Patients with short follow-up (less than 3 months) or with missing data were excluded. We collected all demographic, radiological, and clinical data, including age, sex, comorbidities, ASA score, symptoms, inflammatory syndrome (e.g., fever, C-reactive protein, white blood cells), duration of follow-up, number of episodes and need for surgery.

Recurrence was defined as an episode confirmed by a CT scan with or without hospitalization, confirmed clinically by a physician, or communicated directly to us by the patients or their general practitioners. To complete the follow-up, we conducted telephone interviews using a standardized questionnaire. If patients were unreachable, their general practitioners were contacted to obtain the aforementioned relevant information.

After confirming the patient’s identity, the questions were designed to determine if, following the initial episode of diverticulitis, patients experienced any of the following: recurrences requiring hospitalization, recurrences not necessitating hospitalization, or surgical procedures related to diverticular disease. The complete questionnaire is attached.

### 2.2. Study Endpoints

Our primary endpoint was diverticulitis recurrence. The secondary outcome was the incidence of surgery. A risk factor analysis was performed towards both endpoints.

### 2.3. Statistical Analysis

Descriptive statistics were presented as absolute frequencies for categorical variables and means with standard deviation (SD) for continuous variables. The comparisons of dichotomous values were performed with the chi-square test, while for continuous variables the Student *t*-test was used. Risk factors for recurrence were assessed with a uni- and multivariate regression model, which provided odds ratio (OR) and 95% confidence interval (CI). A Cox regression analysis was carried out to assess the association between covariates and the risk of recurrence during the follow-up. Results were expressed with hazard ratio (HR) and 95%CI. A *p* value < 0.05 was considered statistically significant. The analysis was carried out with MedCalc^®^ Statistical Software version 20.216 (MedCalc Software Ltd., Ostend, Belgium; https://www.medcalc.org; 31 may 2023).

## 3. Results

### 3.1. Patient Inclusion

**Figure 1:**
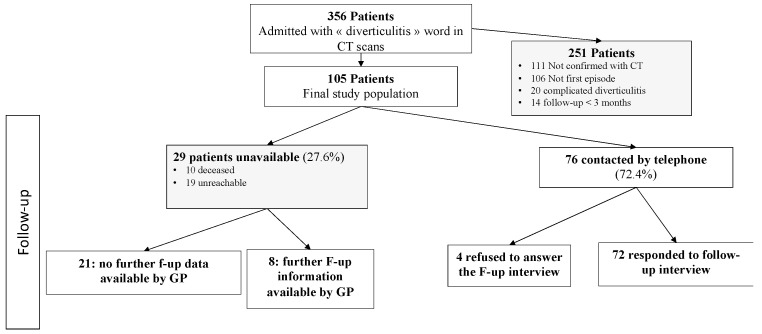
Patient inclusion (consort diagram).

We included 105 patients out of 356 from 1 January 2010 to 30 June 2013. The 251 patients were excluded due to incorrect or unconfirmed diagnosis, prior diverticulitis episode and due to less than 3 months follow-up. As a result, our final study population comprised of 105 patients, who were subsequently analyzed (Figure 1).

### 3.2. Patients’ Characteristics and Follow-Up

Of the 105 included patients, the mean age was 60.9 ± 13.4 years, 62 (59.0%) were female, 35 (33.3%) had at least one comorbidity, and 27 (25.7%) were ASA score ≥ 3. Upon first episode of diverticulitis, patients reported a mean duration of symptoms before entering the emergency department of 3.8 ± 5.4 days. Mean CRP at hospital admission was 90 ± 76 mg/L, and mean white blood cell count was 11.6 ± 3.0 × 10^6^/mL. All patients were treated with antibiotics, 82 (78.1%) were hospitalized.

Regarding follow-up data, 10 (9.5%) patients deceased for reasons unrelated to diverticular disease. Seventy-two (75.8%) were contacted by telephone call and the questionnaire was completed. Four (4.2%) patients refused to answer the questionnaire and 19 (20.0%) could not be reached. For the 29 (27.6%) patients lacking follow-up data in the hospital electronic database, we contacted their general practitioner and relevant follow-up information was provided for 8 of them. The remaining 25 patients, for whom we were unable to gather additional information from their general practitioner, were still included in the study with the data that was available in the hospital’s electronic database, given they had a follow-up period exceeding three months. Therefore, noteworthy follow-up data could be retrieved for all 105 patients. The overall mean follow-up was 116.4 ± 32.2 months. A follow-up longer than 10 years was available for 65 (61.9%) patients.

A thorough analysis of the demographics and patient characteristics divided into groups (recurrence vs. no recurrence, and surgery vs. no surgery) showed no noteworthy difference except a higher prevalence of males among the operated patients (Table 1).

**Figure 2:**
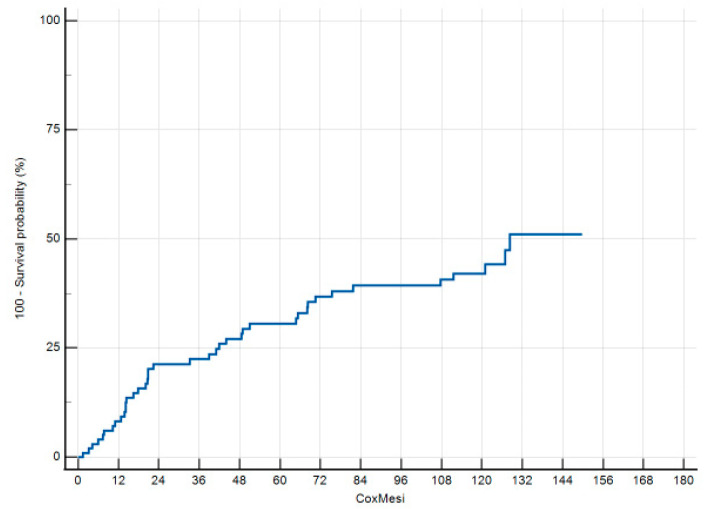
Cox regression analysis.

There were 51 (48.6%) patients with at least one recurrent episode, 11 (21.6%) of them had 4 or more further episodes of acute diverticulitis, while the remaining 40 (78.4%) patients had 1 to 3 recurrences. In the univariate analysis, we identified no factor significantly associated with the risk of recurrence during the follow-up. The cox regression analysis (Figure 2) identified one covariate significantly associated with the risk of recurrence, which was an ASA score ≤ 2 (HR 2.516, 95%CI 1.049–6.031, *p* = 0.039).

In our series, all first episodes of diverticulitis were documented with a CT scan. There was diffuse fat stranding in 14 (13.3%) patients, a small amount of peri-diverticular extra-luminal air in 12 (11.4%) patients, and in 14 (13.3%) patients, the presence of a small amount of free peritoneal fluid. Seven (6.6%) patients were classified CDD 2a (1 cm abscess). None of these signs were significantly correlated with recurrence.

Concerning our second endpoint, we identified 21 (20.0%) patients that eventually required surgery during the follow up period; 12 (57.1%) of them had 1 to 3 recurrences of diverticulitis during the follow-up, while 6 (28.6%) patients had ≥4 episodes of recurrence documented before surgery. All patients were operated in an elective setting and none required emergency surgery. Only three (14.3%) patients were operated without any documented recurrence, but due to ongoing symptomatology. Finally, in the group of patients not requiring surgery, the majority of them (60.7%) had no recurrence. Twenty-eight (33.3%) patients had one to three recurrences and five (6.0%) patients had four or more recurrences which were conservatively treated.

**Figure 3:**
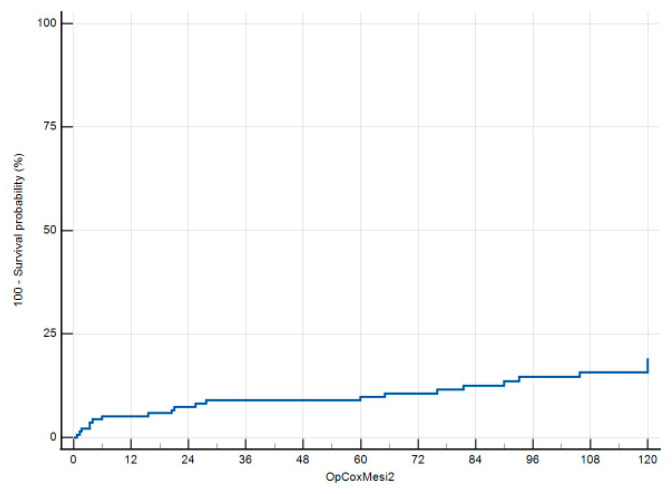
Cox regression analysis.

The univariate analysis (Table 2) for the risk of undergoing surgery during the follow-up showed a significant association with age (OR 1.037, 95%CI 0.999–1.077, *p* = 0.059), and male gender (OR 2.294, 95%CI 1.089–7.874, *p* = 0.033), while CDD grade 1a (OR 0.260, 95%CI 0.056–1.214, *p* = 0.087) was a protective factor compared to stages 1b or 2a. On the multivariate analysis, CDD grade 1a and male gender were confirmed to be positively and negatively associated, respectively, to the risk of surgery. Male gender was confirmed upon Cox regression analysis as a covariate associated with the risk of undergoing surgery during the follow-up (HR 3.111, 95%CI 1.230–7.869, *p* = 0.016) (Figure 3).

## 4. Discussion

Almost half of the patients in our study with a first episode of UD had at least one recurrent episode within a 10-year time frame. This rate is much higher than previously reported with rates varying between 10 and 25% [13,14,15,16]. Systematic reviews that studied the factors responsible for recurrence after the resolution of an uncomplicated episode, reported an overall recurrence rate of 13.3% and an increasing risk of recurrence after every new episode [17]. A recent meta-analysis [14], reported recurrence rates after a first uncomplicated episode ranging from 0.7% to 38.2% but 14 of the 27 studies included had a follow-up of less than 2 years. A study with a 9-year follow up reported recurrence rates of 13.3% [18]. However, this finding may not fully reflect the reality due to the methodology employed in the study, which considered only hospital-admitted cases as recurrences. Given that recurrences treated in an outpatient setting were not taken into consideration, the true recurrence rate could be largely underestimated. In our study, we addressed this limitation by actively seeking information from patients and their treating physicians regarding confirmed episodes of ambulatory-treated diverticulitis. Our higher-than-usual recurrence rate may be attributed to the combination of a long follow-up period within a well-established system of general medical practice that closely follows the general population. Furthermore, the direct telephone contact with the patients and their treating physicians further improved the identification of recurrences and surgical interventions.

Concerning our primary endpoint, we did not identify any risk factors for recurrence. Even signs of a “more complicated” UD, such as CDD 2a stage, free fluid or extra-luminal bubbles, failed to predict recurrence. Recurrence has been shown to increase for patients presenting with an abscess at the initial episode [19,20,21] which drove the ASCRS Guidelines to recommend elective resection after successful non-operative treatment [16]. That correlation was stronger when retroperitoneal abscesses (mHinchey II) were present [22]. The CDD 2a stage is represented by a very small abscess that probably has the same evolution as an uncomplicated episode treated conservatively. Given that all other studies have used the mHinchey classification, there is no actual cut-off value concerning the abscess size, nor a clear topographical localization that precludes or promotes elective surgical treatment.

One out of five patients in our group eventually underwent sigmoidectomy, all in an elective setting, most of them for recurrent episodes or smoldering diverticulitis. Male sex was the only risk factor associated with the need for surgical intervention, in accordance with a recent study [23]. All cases of recurrence were uncomplicated episodes and no patient had to undergo percutaneous drainage or emergency surgery. Three patients underwent elective surgery within the first 6 months after a single first episode for persisting symptoms, attributed to smoldering diverticulitis.

The decision to proceed to an elective surgical treatment after a non-complicated first episode should not be based solely on the number of recurrences. A thorough individual evaluation of each patient`s symptoms, comorbidities, the risk of surgical complications as well as the possibility of a stoma should be made in order to decide the best treatment strategy. In the literature, most emergency colectomies for diverticulitis occur at the initial hospitalization and the likelihood of recurrent hospitalization (5–13%) and emergency colostomy (<5%) is quite low for those who do not have elective resection. Equally important is the finding that elective resection does not eliminate the risk of recurrent diverticulitis, with several studies demonstrating a 5% to 11% risk of recurrence after resection [10,24]. Given that the risk of recurrence increases after each episode, patients may eventually prefer resection to repeated conservative treatments. We believe that this increasing risk, in combination with the long follow up, accounts for our higher than the literature percentage of operated patients. Subsequently it comes as no surprise that more than one third of patients in our group that did not undergo surgery, had presented with up to three recurrent episodes and a small subgroup, more than four.

Our study has limitations. It is a retrospective study, with a relatively small sample size, with all the disadvantages and biases that we tried to counterbalance by providing a thorough inclusion process, a homogeneous group of patients with an accurate identification and classification of their pathology and a very meticulous follow up spanning over a decade. We did not consider racial differences, given that our final database consisted almost totally of local patients, Caucasians, indigenous to the Italian-speaking Canton of Switzerland and northern Italy. This follow up was completed with three input pathways, the electronic data systems, general practitioners and the patients themselves, thus including data generally missed in similar studies, as for example commonly occurring outpatient visits for diverticulitis. The follow-up may have suffered from recall bias (underestimating true incidence) and the imprecision of diagnosis, as we could not verify all outpatient diagnosis of diverticulitis (overestimating true incidence). Similarly, the surgical indication could not be verified on all aspects retrospectively, as it was sometimes based on surgeon’s preference and therefore could lack external validity.

All our patients had an uneventful course during the acute setting and none required emergency surgery or interventional radiology treatment, despite the presence of “complicated” features on CT scans, like extraluminal air (close to the bowel wall), small amounts of free liquid, or small abscesses (up to 1 cm) in some patients. None of these features were predictive of recurrence. We therefore believe that the definition of “uncomplicated” diverticulitis should include all patients with diverticulitis except those with large (>3 cm) abscesses that require percutaneous drainage, or those with diffuse peritonitis with free air and liquid [16]. Most likely, within this group of UD, a subgroup of patients may require antibiotic treatment (those with “worrisome features”, on CT-scan or on clinical assessment), whereas others do not. During the time period of the study (ten years ago), it was standard practice to treat all such patients with antibiotics and frequently admit them to the hospital. Therefore, we were unable to examine the value of the classification systems or CT-scan features to differentiate which patients could be easily treated without antibiotics and/or in an outpatient setting, as suggested by more recent evidence [8,25,26]. A simple, treatment-oriented classification system is proposed in Table 3.

## 5. Conclusions

After a decade of follow-up, half the patients experienced recurrence after an uncomplicated first episode and 20% of them benefitted from elective sigmoidectomy for multiple recurrent episodes or persistent symptoms. Worrisome features on CT scan, such as micro-abscess, moderate free fluid or air bubbles, did not affect recurrence or the need for surgery, raising the need for an updated definition of uncomplicated diverticulitis that might potentially improve clinical management.

## Figures and Tables

**Table 1:** Demographics and patient characteristics.

	Patients with Recurrence N = 51	Patients without Recurrence N = 54	*p*	Patients Operated N = 21	Patients Not Operated N = 84	*p*
Age, years (SD)	60.2 (13.9)	61.6 (12.9)	0.612	55.9 (12.7)	62.2 (13.3)	0.055
Gender, female (%)	30 (58.8)	32 (59.3)	0.964	8 (38.1)	54 (64.3)	0.030
ASA score						
1, n (%)	18 (35.3)	19 (35.2)	0.198	7 (33.3)	30 (35.7)	0.373
2, n (%)	24 (47.1)	17 (31.5)		11 (52.4)	30 (35.7)	
3, n (%)	7 (13.7)	16 (29.6)		2 (9.5)	21 (25.0)	
4, n (%)	2 (3.9)	2 (3.7)		1 (4.8)	3 (3.6)	
Comorbidities, n (%)						
• Pulmonary disease, n (%)	2 (3.9)	2 (3.7)	0.954	0	4 (4.8)	0.310
• Diabetes mellitus, n (%)	1 (2.0)	4 (7.4)	0.192	0	5 (6.0)	0.254
• Cardiological disease, n (%)	6 (11.8)	10 (18.5)	0.338	2 (9.5)	14 (16.7)	0.417
• Hypertension, n (%)	14 (27.5)	18 (33.3)	0.515	5 (23.8)	27 (32.1)	0.460
Symptoms duration, days (SD)	3.6 (4.7)	3.9 (6.0)	0.739	3.3 (3.3)	3.9 (5.8)	0.679
Peri diverticular fat stranding (vs. diffuse), n (%)	8 (15.7)	6 (11.1)	0.493	3 (14.3)	11 (13.1)	0.886
Presence of extraluminal air, n (%)	6 (11.8)	6 (11.1)	0.912	2 (9.5)	10 (11.9)	0.760
Presence of peritoneal fluid, n (%)	6 (11.8)	8 (14.8)	0.647	4 (19.0)	10 (11.9)	0.391
CDD classification						
• 1a	13 (25.5)	15 (27.8)	0.905	2 (9.5)	26 (31.0)	0.063
• 1b	35 (68.6)	35 (64.8)		16 (76.2)	54 (64.3)	
• 2a	3 (5.9)	4 (7.4)		3 (14.3)	4 (4.8)	
Temperature ≥ 38.5 °C, n (%)	8 (15.7)	7 (13.0)	0.692	1 (4.8)	14 (16.7)	0.165
CRP, mg/L (SD)	98 (86)	82 (64)	0.263	87 (59)	90 (80)	0.862
White blood cell count, ×10^9^/L (SD)	11.4 (2.9)	11.7 (3.0)	0.620	12.4 (10.3)	11.4 (2.9)	0.189
Follow-up duration, months (SD)	113.7 (37.2)	118.9 (26.7)	0.410	120.5 (38.2)	115.4 (30.7)	0.517
Number of recurrences						
• 0, n (%)	0	54 (100)	-	3 (14.3)	51 (60.7)	-
• 1–3, n (%)	40 (78.4)	0		12 (57.1)	28 (33.3)	
• ≥4, n (%)	11 (21.6)	0		6 (28.6)	5 (6.0)	

**Table 2:** Uni- and multivariate analyses for factors associated with sigmoidectomy.

	Univariate Analysis (OR and (95% CI)	*p*	Multivariate Analysis (OR and (95% CI)	*p*
Age, years	1.037 (0.999–1.077)	0.059	1.021 (0.981–1.063)	0.312
Gender, female	0.342 (0.127–0.918)	0.033	0.301 (0.109–0.834)	0.021
ASA score ≥ 3 pt	1.164 (0.654–2.072)	0.605		
Comorbidities, n (%)				
• Pulmonary disease, n (%)	1.266 (0.140–11.452)	0.834		
• Diabetes mellitus, n (%)	1.538 (0.175–13.519)	0.678		
• Cardiological disease, n (%)	1.900 (0.397–9.096)	0.422		
• Hypertension, n (%)	1.516 (0.503–4.570)	0.460		
Symptoms duration, days	1.024 (0.917–1.142)	0.678		
Peri diverticular fat stranding (vs. diffuse)	0.904 (0.228–3.583)	0.886		
Presence of extraluminal air	1.284 (0.259–6.357)	0.759		
Presence of peritoneal fluid	0.574 (0.161–2.053)	0.393		
CDD classification				
• 1a	0.260 (0.055–1.214)	0.087	0.201 (0.042–0.957)	0.044
• 1b	Ref.	-	Ref.	-
• 2a	2.532 (0.512–12.500)	0.254		
Temperature ≥ 38.5 °C	4.000 (0.495–32.300)	0.193		
CRP, mg/L	1.001 (0.994–1.007)	0.860		
White blood cell count, ×10^9^/L	0.901 (0.770–1.054)	0.191		

**Table 3:** Classification proposal.

Acute	Uncomplicated diverticulitis Without worrisome features With worrisome features (*) Diverticulitis with abscess requiring drainage Diverticulitis with clinical diffuse peritonitis Air/purulent only Fecal regional Fecal diffuse
Chronic	Multi-recurrent uncomplicated diverticulitis Smoldering diverticulitis Fistulizing diverticulitis Obstructing diverticulitis
* Worrisome features: CT-scan: extraluminal air-bubbles, free peritoneal liquid, extensive phlegmon Clinical: T > 39, CRP > 150 mg/L, signs of sepsis, immunosuppression, diabetes.	

## Data Availability

Data is unavailable due to privacy restrictions.
